# Bibliometric analysis on exploitation of biogenic gold and silver nanoparticles in breast, ovarian and cervical cancer therapy

**DOI:** 10.3389/fphar.2022.1035769

**Published:** 2022-12-22

**Authors:** Meena Bhandari, Seema Raj, Ashwani Kumar, Dilraj Preet Kaur

**Affiliations:** ^1^ Department of Chemistry, School of Basic and Applied Sciences, K.R Mangalam University, Gurugram, India; ^2^ Department of Computer Sciences, School of Engineering and Technology, K.R Mangalam University, Gurugram, India; ^3^ Department of Physics, School of Basic and Applied Sciences, K.R Mangalam University, Gurugram, India

**Keywords:** biogenic, silver nanoparticles, gold nanoparticles, cancer therapy, bibliometric analysis

## Abstract

Multifunctional nanoparticles are being formulated to overcome the side effects associated with anticancer drugs as well as conventional drug delivery systems. Cancer therapy has gained the advancement due to various pragmatic approaches with better treatment outcomes. The metal nanostructures such as gold and silver nanoparticles accessible *via* eco-friendly method provide amazing characteristics in the field of diagnosis and therapy towards cancer diseases. The environmental friendly approach has been proposed as a substitute to minimize the use of hazardous compounds associated in chemical synthesis of nanoparticles. In this attempt, researchers have used various microbes, and plant-based agents as reducing agents. In the last 2 decades various papers have been published emphasizing the benefits of the eco-friendly approach and advantages over the traditional method in the cancer therapy. Despite of various reports and published research papers, eco-based nanoparticles do not seem to find a way to clinical translation for cancer treatment. Present review enumerates the bibliometric data on biogenic silver and gold nanoparticles from Clarivate Analytics Web of Science (WoS) and Scopus for the duration 2010 to 2022 for cancer treatment with a special emphasis on breast, ovarian and cervical cancer. Furthermore, this review covers the recent advances in this area of research and also highlights the obstacles in the journey of biogenic nanodrug from clinic to market.

## 1 Introduction

Cancer in general is a highly complex disease prevalent globally and incidents of breast cancer in women are on the rise, 30% new cases are reported every year (Holliday and Spiers, 2011). Chemotherapy, radiotherapy, immunotherapy, surgery, vaccinations for cancer, stem cell therapy, photo dynamic therapy and combinational methods are applied for cancer treatment which pose their own risk as toxicity, limited bioavailability, non-specific action, etc. (Raza et al., 2016; Chahal et al., 2018).

Recent advances in nanotechnology may pose an alternative approach to treat cancer as high surface area, shape and charge on the nanoparticles help them to interact with biological membrane, indicate increase in permeability, and thus enhance their activity (Klochkov et al., 2021). Gold nanoparticles (Au@NP)and silver nanoparticles (Ag@NP) have revealed their remarkable possibilities (Sarkar et al., 2011 and Sarkar et al., 2012) for diagnosis and treatment of cancer and as drug delivery in both *in vivo*, *in vitro* systems (Cai et al., 2008) and could minimize the chemotherapeutic drug-induced antagonistic effects. Biological synthesis of nanomaterials is prominently acquiring importance as physical and chemical methods employed for synthesis of nanoparticles demonstrate few limitations as high cost, low-productivity and toxicity (Acharya and Sarkar 2014; Mollick et al., 2014; Jadhav et al., 2018). Nano biomaterials have been reported to be approved by FDA for use as anticancer drugs, and diagnostic agents (Anand et al., 2021).

Bioactive phytochemicals such as polyphenols, phenolic acids, flavonoids, terpenes, and alkaloids etc. present in plants have been shown to exhibit anticancerous properties (Ren et al., 2003; Balunas and Kinghorn, 2005; Hu, 2011). Generally phytoconstituents present in the plants are converted to nanoparticles by reaction with metal salts which behave as reducing and capping agents (Singh P et al., 2018) Figure 1, but researchers also used other extended methods of preparation of such nanoparticles for better yield. Nano particles act as tug boats of research to overcome the above listed challenges (Hira et al., 2018). Since different plants possess different phytoconstituents, having variable chemical structure, display variable actions against different types of cancers. The action of nanoparticles on target cancer cell is represented in Figure 2.

**FIGURE 1:**
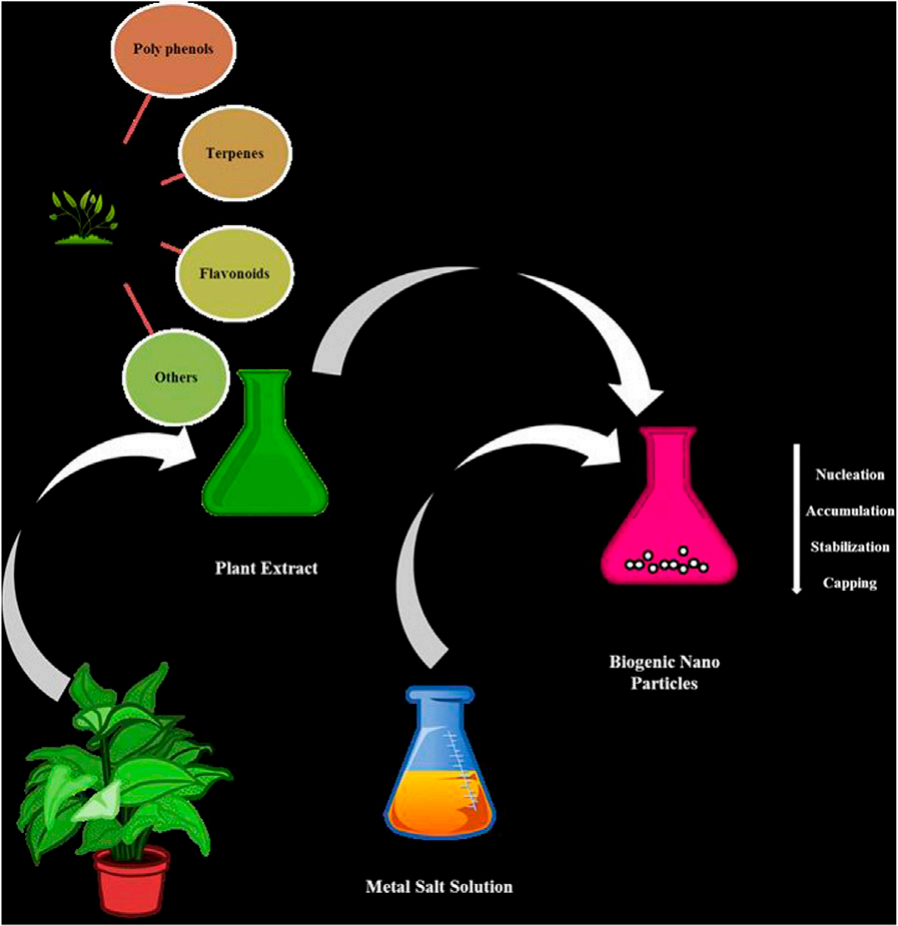
General green synthesis of nanoparticles by using plant extracts and metal salt solutions.

**FIGURE 2:**
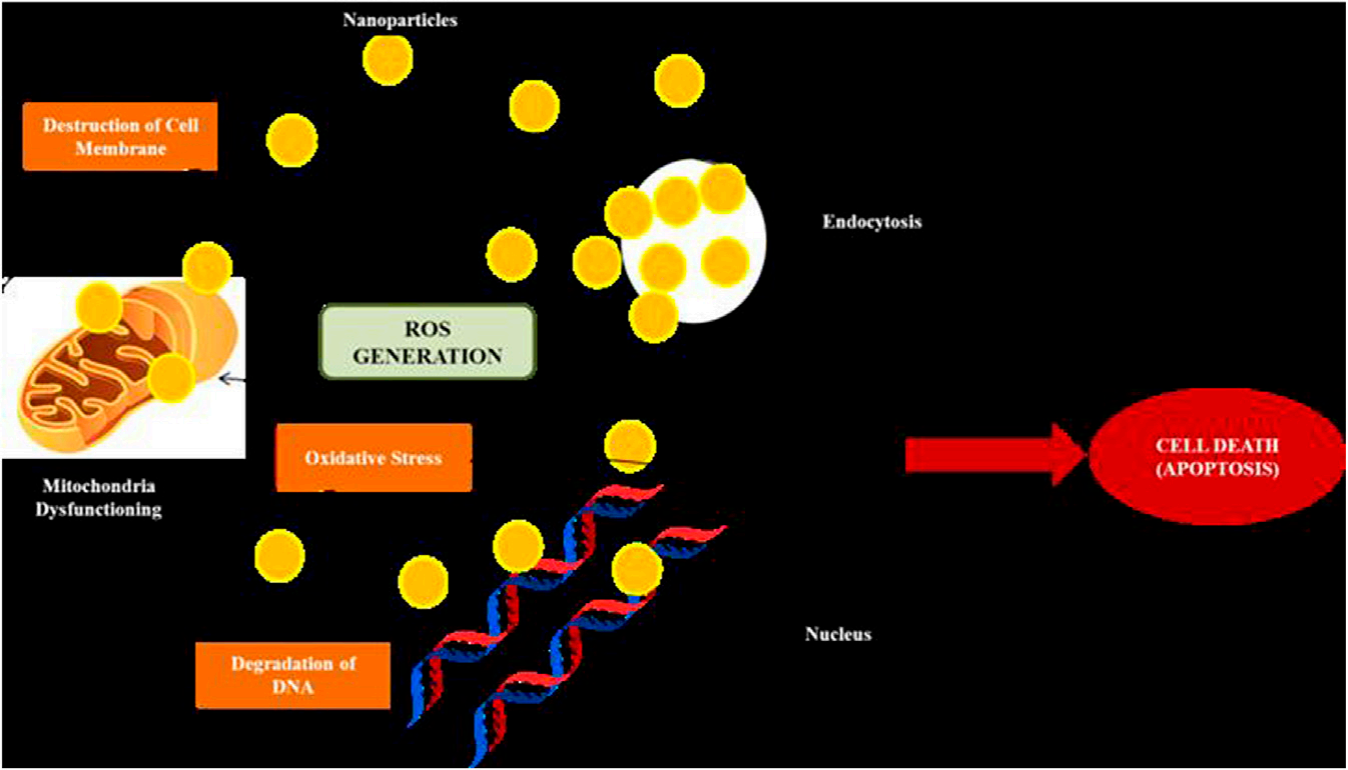
Mechanism of action of nanoparticles on cancer cell.

The combination of nanotechnology and pharmaceutical sciences creates a boom in the medical field due to a variety of applications. Nanoparticles are less toxic, possess better optical, photothermal properties and can be used as biomedicine (Raza et al., 2016; Yazdi et al., 2016), assay for diagnostic (Liu et al., 2015), drug delivery or gene delivery (Yang et al., 2015) and expansion of radiotherapy. AuNPs have shown efficacy for treatment of prostate (Barabadi et al., 2019), breast (Anand et al., 2021), and colorectal cancer (Khan A et al., 2021). Nowadays, biogenic nanoparticles, prepared by natural extracts like plant extracts are in the frame for use as treatment for cancer as a sustainable approach. Some people used olive oil, licorice root (*Glycyrrhiza glabra*) and coconut oil for preparation of Gold coated Iron nanoparticles (Al-Radadi, 2022). Not only in cancer, the role of biogenic Au@NP and Ag@NP is magnificent in other fields as well. Studies on mice show *in vivo* anti-diabetic and anticancer effects as well anti-hypoglycemia and hepatic-protective potential when biogenic Au@NP and Ag@NP prepared from *Ajuga bracteosa*, aqueous extract were used (Nazer et al., 2022).

Though the utilization of natural resources or molecules to treat human malignancies, illness, diseases, is expanding (Tuasha et al., 2018; Kashyap et al., 2021) as they play an important role as chemo-protective or radio-protective and therapeutic agents to treat cancer (Khan F. A et al., 2021; Raj et al., 2022) but it is also observed that because of chemical and physical changes in the process, the alteration in the molecular structure of phytochemicals may alter their efficacy against cancer (Seca and Pinto, 2018). Such challenges emphasise the requirement for different approaches to overcome these constraints. Consequently, creating innovative formulations and nanotechnology-based methods for delivering drugs are considered to be the promising approach.

## 2 Quantitative approach and strategy of bibliometric analysis

Apart from obstacles, nanoparticles are still winning on other methods of cancer therapy. Among various applications of nanoparticles, the large quantity of drug delivery with large surface area of nanoparticle is inspiring (Faraji and Wipf 2009).

This paper emphasizes on bibliometric analysis of researches undergoing in the field of use of biogenic nanoparticles in cancer treatment, specifically breast, ovarian and cervical cancer. Such analysis focuses on publication trend in biogenic nanoparticle cancer therapy.

Bibliometric is the application of statistical techniques for assessing the research output of nations, institutions, and people, as well as quantifying academic success (Darroudi et al., 2021). However, bibliometric analysis is insufficient in evaluating a study area’s outputs; therefore, it needs to be taken in to consideration other types of inputs, including a literature review to learn about trends in publishing. This study examines the state of research in this area of study through bibliometric and qualitative literature reviews from the past to the present. This section provides an overview of the current status and future directions in this field.

For bibliometric analysis, data is collected on biogenic silver and gold nanoparticles from Clarivate Analytics Web of Science (WoS) and Scopus for the duration 2010 to 2022 for cancer treatment with a special emphasis on breast, ovarian and cervical cancer. The core collected data was 341 papers, after searching the keywords as “Biogenic gold nanoparticles + breast cancer + clinical trials”, “Biogenic gold nanoparticles + ovarian cancer + clinical trials”, “Biogenic gold nanoparticles+ cervical cancer + clinical trials”, “Biogenic gold nanoparticles+ cancer therapy + clinical trials”, “Biogenic silver nanoparticles + breast cancer + clinical trials”, “Biogenic silver nanoparticles + ovarian cancer +clinical trials”, “Biogenic silver nanoparticles + cervical cancer + clinical trials” and “Biogenic silver nanoparticles + cancer therapy + clinical trials”. The duplicity of publication was found in each keyword search; therefore after removing duplicity, 141 papers were left. These 141 articles were analysed on the parameters indicated:

• General publication trend
• Most cited papers
• Most Contributing authors
• Top Journals
• Articles on Clinical trials
• Plant extract for Biogenic Au@NP and Ag@NP

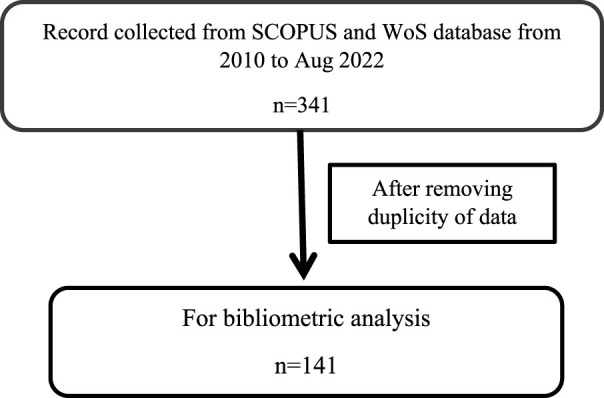


## 3 Result and discussion

It was observed that out of 341, 141 publications related to biogenic gold and silver nanoparticles for identified cancer therapies. This bibliographic analysis provides the idea general publication trends in this field, the most contributing authors with citation levels, top groups which are working in collaborations, different plant extracts used to formulate biogenic nanoparticles, applications of nanoparticles on different cell lines and obstacles in the journey of Nanoparticles from clinic to market.

### 3.1 General publication trend

According to Table 1, the maximum 42 publications were reported in 2021 but the maximum citations were observed for papers published in about 2018 though publications number is very less i.e., 13.

**TABLE 1:** Details of number of publications in each year.

Year	Number of publications	Average number of authors for each document
2013	2	7.00
2014	2	3.50
2015	3	9.33
2016	9	3.78
2017	7	4.14
2018	13	5.69
2019	17	4.06
2020	19	5.37
2021	42	5.88
2022	27	6.85

The publications illustrate a variety of applications of biogenic Ag@NP and Au@NP with reference to cancer therapy. Currently, nanoparticles are used to improve impact of blue light therapy for cancer treatment. Blue Light therapy is a therapy to heal deep wounds (Akasov et al., 2022). As previously discussed, ecological and green synthetic approach to the synthesis of metal nanoparticles has gained a lot of importance in the recent era, like gold coated iron (Fe@Au) nanoparticles have been synthesized using an extract solution of olive oil, licorice root and coconut oil (Al-Radadi, 2022). It was suggested that Au@NP synthesized from biocompatible plants play vital role in cancer therapy. Researchers used Fluorescent plant based markers with biogenic Au@NP for cancer detection as well (Sargazi et al., 2022). In some studies, biogenic Au@NP was used for neurological controls as well (Zare et al., 2022). As flavonoglycone hesperidin, an anticancer agent has poor biocompatibility i.e. hesperidin loaded Au@NP were used due to their bioactive potential and compatibility (Sulaiman et al., 2022).

Specifically, biogenic Ag@NP and Au@NP have attracted attention for their antineoplastic activity toward leukaemia attributable to their unique physico-chemical properties (Mostafavi et al., 2022). Biogenic Ag@NP can be used in dental therapy due to its antibacterial and antifouling properties, the scope of such work also relates to mouth cancer (Ahmed et al., 2022). Researchers used biogenic Ag@NP which displayed potent toxic effects against both cancer cell lines and pathogens, and also exhibited antioxidant activity (Wei et al., 2021). Recently Ag@NP were also tested with spirulina on rats to treat prostate cancer, and it was found that a class of rats showed good result in the context of treatment with stability in hormone imbalance (El-Magid et al., 2022).

In 2021, maximum publications were reported, but most of them were review articles related to cancer therapy Au@NP synthesized using *Argemone mexicana* L. aqueous extract have been used on cell line, HCT-15. It was found that these biogenic nanoparticles were effective in cell growth suppression and induction of apoptosis (Datkhile et al., 2021). As an anti-leukaemia agent, Au@NPs prepared by using *Tribulus terrestris* extract, were used and it was observed that the Au@NPs exhibited steady reduction in % cell viability with an increase in its concentration (Zhao et al., 2021). It was reported that cetuximab-targeted gold nanorods (CTX-AuNR) is an attractive therapeutic strategy for triple negative breast cancer (TNBC). CTX-AuNR with near infrared (NIR) irradiation, can serve as a potent photoimmunotherapy strategy for handling epidermal growth factor receptor overexpressing TNBC cells (Emami et al., 2021). The Nutraceuticals present in marine algae is also found to show spectacular algal-mediated synthesis of nanotheranostics. Such algae used to prepare thermodynamically stable NPs (Menaa et al., 2021). The phytochemicals present in Pomegranate (*Punica granatum* L.) has demonstrated tremendous potential for preventing cancer. The chemical components of pomegranates, the outcomes of preclinical (*in vitro*, *ex vivo*, and *in vivo*) and clinical investigations on the anticancer impact of pomegranate phytochemicals and molecular targets in a variety of malignancies, such as breast cancer, gastrointestinal tract cancer, uterine and ovarian cancer, leukemia, lung cancer, neurological (glioma) etc. have been published (Wong et al., 2021).

Recent developments in determining the half-maximal inhibitory concentration, a crucial factor for advancing clinical trials, have mostly focused on the use of standard 2D cell culture and *in vivo* murine models to assess the anticancer effect of green biogenic Au and Ag nanoparticles (Tinajero-Díaz et al., 2021).

According to bibliographic analysis the most cited papers were reported in 2018. There are two most cited research articles, one is from “International Journal of Molecular Sciences” and other is from “Frontiers in Microbiology”. The first paper emphasized on the supreme role of Au@NPs in the diagnosis of a variety of cancers and for drug delivery applications. Gold nanoparticles non-toxic and non-immunogenic properties, as well as their high permeability and retention, impact, offer further advantages by making it simple for medications to accumulate at tumour locations (Singh J et al., 2018). The advantages of biogenic nanoparticles over other antibacterial or antimicrobial therapies were also discussed, which revealed their potential and non-toxic behaviour (Baptista et al., 2018).

### 3.2 Most cited papers

Bibliographic analysis of required data emphasize on applications of Au@NP in diagnosis and as therapeutic agent for human cancer due to its spectacular physical properties. The other published articles deals with the introduction, reviews and applications of Nanoparticles (Table 2). The usage of biogenic nanoparticles (Ag and Au) offers a current approach which not only leads to green synthesis or use of plant extract, but also overcome the toxic effects of nanomaterials as well. Researchers used such nanoparticles *in vivo*, *in vitro* and *ex vitro* strategies and confirmed the benefits of such biogenic nanoparticles in cancer therapy.

**TABLE 2:** Details of most cited papers till date.

Authors	Title	Year	Source title	References
Singh P., Pandit S., Mokkapati V.R.S.S., Garg A., Ravikumar V., Mijakovic I	Gold nanoparticles in diagnostics and therapeutics for human cancer	2018	International Journal of Molecular Sciences	Singh J et al. (2018)
Baptista P.V., McCusker M.P., Carvalho A., Ferreira D.A., Mohan N.M., Martins M., Fernandes A.R	Nano-strategies to fight multidrug resistant bacteria-"A Battle of the Titans"	2018	Frontiers in Microbiology	Baptista et al. (2018)
Rafique M., Sadaf I., Rafique M.S., Tahir M.B	A review on green synthesis of silver nanoparticles and their applications	2017	Artificial Cells, Nanomedicine and Biotechnology	Rafique et al. (2017)
Jeyaraj M., Sathishkumar G., Sivanandhan G., MubarakAli D., Rajesh M., Arun R., Kapildev G., Manickavasagam M., Thajuddin N., Premkumar K., Ganapathi A	Biogenic silver nanoparticles for cancer treatment: An experimental report	2013	Colloids and Surfaces B: Biointerfaces	Jeyaraj et al. (2013)
Austin L.A., MacKey M.A., Dreaden E.C., El-Sayed M.A	The optical, photothermal, and facile surface chemical properties of gold and silver nanoparticles in biodiagnostics, therapy, and drug delivery	2014	Archives of Toxicology	Austin et al. (2014)
Bahrami B., Hojjat-Farsangi M., Mohammadi H., Anvari E., Ghalamfarsa G., Yousefi M., Jadidi-Niaragh F	Nanoparticles and targeted drug delivery in cancer therapy	2017	Immunology Letters	Bahrami et al. (2017)
Navya P.N., Kaphle A., Srinivas S.P., Bhargava S.K., Rotello V.M., Daima H.K	Current trends and challenges in cancer management and therapy using designer nanomaterials	2019	Nano Convergence	Navya et al. (2019)
Prasad M., Lambe U.P., Brar B., Shah I., J M, Ranjan K., Rao R., Kumar S., Mahant S., Khurana S.K., Iqbal H.M.N., Dhama K., Misri J., Prasad G	Nanotherapeutics: An insight into healthcare and multi-dimensional applications in medical sector of the modern world	2018	Biomedicine and Pharmacotherapy	Prasad et al. (2018)
Carvalho P.M., Felício M.R., Santos N.C., Gonçalves S., Domingues M.M	Application of light scattering techniques to nanoparticle characterization and development	2018	Frontiers in Chemistry	Carvalho et al. (2018)
Padovani G.C., Feitosa V.P., Sauro S., Tay F.R., Durán G., Paula A.J., Durán N	Advances in Dental Materials through Nanotechnology: Facts, Perspectives and Toxicological Aspects	2015	Trends in Biotechnology	Padovani et al. (2015)

### 3.3 Most contributing author

**FIGURE 3:**
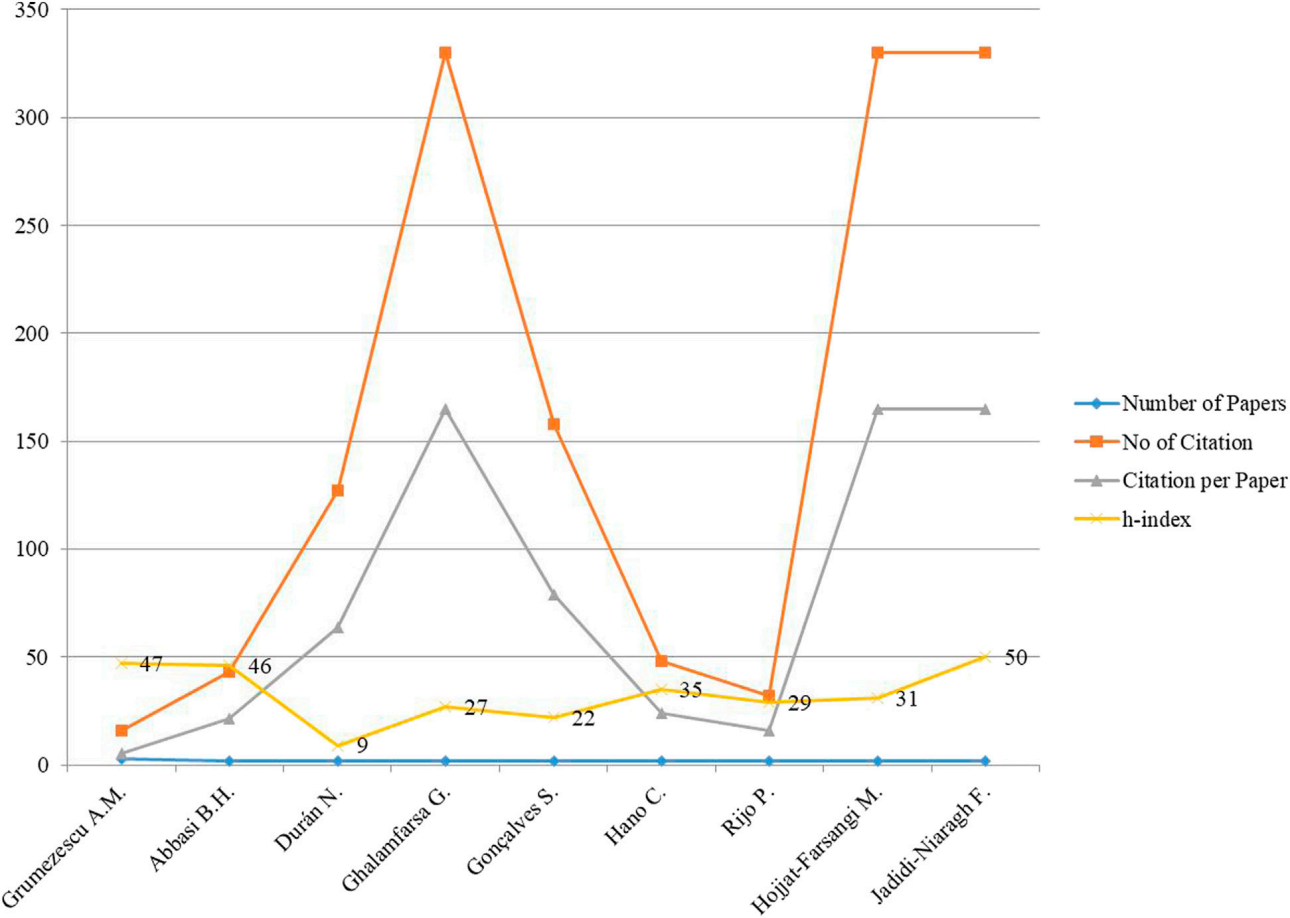
Author details in cancer therapy with nanoparticles.

The bibliographic analysis showed the Dr Alexandru Mihai Grumezescu, Romania, contributed the maximum on applications nanomaterials as anticancer and antimicrobial activities. Figure 3 is shows that though numbers of publications in this field are less but the citation factor is high. Researchers who are working in these fields have good h-index as well, in the range of 20–50.

### 3.4 Top journals

As per data we have identified six top Journals in the required key words projected for bibliographic analysis are Pharmaceutics, Advanced Drug Delivery Reviews, Nanomaterials, International Journal of Molecular Sciences, International Journal of Nanomedicine and Nanoscale consecutively.

### 3.5 Application of Au@NPs and Ag@NPs on variety of cell line

Several anti-cancer drugs such as Myocet, DaunoXome, ONPATTRO Patisiran ALN-TTR02, VYXEOS CPX-351 based on nanotechnology have been approved by US FDA and EMA (Bhardwaj et al., 2021).

The efficiency of silver and gold nanoparticles derived from *Acalypha*. indica leaves were tested for their cytotoxicity against MDA-MB-231, human breast cancer cells and they illustrated 40% cell inhibition and exhibited prominent cytotoxic and apoptotic effect through DNA destruction (Krishnaraj et al., 2014).

The gold nanoparticles biosynthesized using *Argemone mexicana* L. aqueous extract have been found to show genotoxic effects on human cervical adenocarcinoma (HeLa), human breast adenocarcinoma (MCF-7) and human colon adenocarcinoma (HCT-15) cells by stopping growth of cell and initiation of caspase-mediated apoptosis by stimulating p53 and caspase-3 genes generation (Datkhile et al., 2021). Gold nanoparticles (AuNPs) prepared from Mimosa pudica leaves extract exhibited anticancer properties for breast cancer cell lines (MDA-MB-231 & MCF-7) without creating any toxicity. The cytotoxicity test was done by fluorescence microscopy on cancer cells and Human mammary epithelial cells, it was found that there is no toxic effect on normal cells (Suganya et al., 2016). In most of the cases, different breast cancer cell line like MDA-MB-468, MCF-7, MDA-MB and MDA-MB-231 were used to evaluate the anti-cancer efficacy of silver and gold nanoparticles. AuNPs from *Commiphora wightii* leaf extract were prepared which displayed anticancer properties on MCF-7 cells by killing them (Uzma et al., 2020). SPE-AuNPs were prepared from the aqueous peel extract of *Spondias dulcis (SPE)* which displayed substantial cytotoxicity to MCF-7 human breast cancer cells through the production of reactive oxygen species (Pechyen et al., 2022).

Curcumin-coated silver nanoparticles (cAgNPs) along with cisplastin showed better efficacy through programmed cell death in A2780 cells as compared to when cAgNPs or cisplastin are used alone (Ramezani et al., 2019). The viability of MCF-7 cells was reduced to 50% with AgNPs synthesized from *Dendrophthoe falcata* (Sathishkumar et al., 2014). AgNPs synthesized from *Datura inoxia* impedes 50% propagation of human breast cancer cell line MCF7 (Gajendran et al., 2014). Cell viability of MCF-7 cells effectively decreased when treated with *Rhizophora mucronata* mediated Ag NPs (Rajivgandhi et al., 2022a). Efficacy of Silver nanoparticles, synthesized from the extracts of *Geodorum densiflorum* rhizome, *Kaempferia rotunda* and *Zizyphus mauritiana* was determined against GSC cells along with MCF-7 and BxPC-3 cells and *in vivo* anticancer properties were studied against EAC cells (Kabir et al., 2022) and *G. desiflorum* nanoparticles were found to inhibit growth of EAC cells by 90%.

Cytotoxicity studies of Ag/AgCl-NPs obtained from the *Z. mauritiana* fruit extract were monitored against human breast cancer cell line (MCF-7) and mice Ehrlich ascites carcinoma (EAC) cells and it was observed that apoptosis is induced through the Fas-mediated pathway (Kabir et al., 2020).

Biogenic AuNPs derived from *Anacardium occidentale* leaves showed IC50 of 6 and 600 μg/ml, respectively towards MCF-7 cells and peripheral blood mononuclear cells (PBMCs) using an MTT assay (Sunderam et al., 2019). Biogenic AuNPs prepared from C. papaya and *Catharanthus roseus* (C. roseus) exhibited cytotoxicity against MCF-7 (Muthukumar et al., 2016). Cytotoxic impact of AuNPs prepared from *Aegle marmelos, Eugenia jambolana,* and *Soursop* were evaluated against MCF-7 cells (Vijayakumar, 2019) having IC 50 values of 172 ± 4, 163 ± 4, and 98 ± 4 µg/ml, respectively. Au@NPs synthesized from Dragon fruit were found to exhibit cytotoxicity having IC50 value of around 500 μg/ml against MCF-7 breast cancer cells using an alamarBlue^®^ assay (Divakaran et al., 2019).

Nerium oleander-conjugated gold nanoparticles prepared from stem bark of the plant showed cytotoxicity against MCF-7 breast cancer cell. It exhibited efficacy for the apoptosis of tumor selectively (Barai et al., 2018). The Lycium chinense AgNPs synthesized from fruit extract of the plant showed appreciable cytotoxic impact against human breast cancer MCF7 cell line (Chokkalingam et al., 2019). Green AuNPs prepared from *Camellia sinensis*, *Coriandrum sativum*, *Mentha arvensis*, *Phyl-lanthus amarus*, *Artabotrys hexapetalus*, *Mimusops elengi*, *Syzygium aromaticum* exhibited anticancer effectiveness against MCF7 with minimum concentration of 2 μg/ml (Kamla Priya and Iyer, 2015).

### 3.6 Plant extract for Biogenic Au@NP (gold nanoparticles) and Ag@NP (silver nanoparticles)

Table 3 represents the plants which have been utilized for the preparation of nanoparticles for cancer therapy. According to the studies Licorice root (*Glycyrrhiza glabra*), *Medicago sativa, Olax Scandens, Hubertia ambavilla*, *Hypericum lanceolatum, Argemone mexicana L., Tribulus terrestris,* Solanaceae family, Marine algae, *Carissa carandas* leaf, *Leucophyllum frutescens* and *Russelia equisetiformis* leaves, *Eucalyptus tereticornis* leaves, *Artemisia Sieberi Besser* leaves, *Gelidiella acerosaon, Ajuga bracteosa,* Floral extracts of *Callistemon viminalis, Caesalpinia pulcherrima* stem, *Garcinia mangostana, Cinnamomum zeylanicum, Salvia officinalis* leaf, *Tilia cordata* flowers, *Aegle marmelos, Curcumae kwangsiensis* leaf, *Spondias dulcis* (*Anacardiaceae*) peel, *Vigna radiate* and many more, plant part extracts were used to prepare biogenic gold and silver nanoparticles for different applications. It implies that the scope of biogenic nanoparticles is better in the field of cancer therapy.

**TABLE 3:** Plant used to form gold and silver nanoparticles along with their applications.

S.No.	Name of plants to prepare biogenic nanoparticles	Type of nanoparticles	Applications	Reference
1	Olive oil, Licorice root (*Glycyrrhiza glabra*) and Coconut oil	Gold coated iron (Fe@Au) nanoparticles	Against *Helicobacter pylori* (H. pylori) and ulcer	Al-Radadi, (2022)
2	*Medicago sativa, Olax Scandens, H. ambavilla*, and *H. lanceolatum*	Fluorescent-plant-based markers, including Au@NPs	Cancer therapy	Sargazi et al. (2022)
3	*Argemone mexicana L*	*Argemone mexicana* –Au@NPs	Human colon cancer cell line, HCT-15	Datkhile et al. (2021)
4	*Tribulus terrestris*	Au@NPs-Tribulus	Potential anti-Leukemia drug	Zhao et al. (2021)
5	Solanaceae family	Nanoparticles	Chemotherapeutic agents in various *in vitro* and *in vivo* cancer models	Kowalczyk et al. (2022)
6	*Spirulina* algae to AgNPs as a combination	Ag@NPs and Spirulina algae (Sp)	Prostate cancer	El-Magid et al. (2022)
7	Marine algae	Nanoparticles	Nanotheranostic purposes	Menaa et al. (2021)
8	*Carissa carandas* leaf	Ag@NP	Antimicrobial activity and Antibiofilm activity	Rahuman et al. (2021)
9	*Leucophyllum frutescens* and *Russelia equisetiformis* leaves	Ag@NP	Potential cytotoxic and Antibacterial capacity	Mohammed and Al-mergin, (2021)
10	*Eucalyptus tereticornis* leaves	Ag@NP	Potential anticancer activity	Kiran et al. (2020)
11	*Artemisia Sieberi Besser* leaves	Ag@NP	Stability checking of Nanoparticles at different parameters	Rousta and Ghasemi, (2019)
12	Red algae *Gelidiella acerosaon*	-	Anticancer properties	SM et al. (2018)
13	*Ajuga bracteosa*	AB-Ag@NP	Anti-diabetic and hepato-protective phytoconstituents	Nazer et al. (2022)
14	Floral extracts of *Callistemon viminalis*	Nickel oxide Nanoparticles (NiO-NP)	Diverse biomedical applications	Sani et al. (2021)
15	Marine Algae *Chaetomorpha linum*	Ag@NP	Human Colon Cancer Cell HCT-116	Acharya et al. (2021)
16	*Caesalpinia pulcherrima* stem	Ag@NP	Breast cancer cells through *in vitro* approaches	Rajivgandhi et al., (2022b)
17	*Garcinia mangostana*	Ag@NP	Against human breast cancer	Alobaid et al. (2022)
18	*Cinnamomum zeylanicum*	Ag@NP	Improve the fertility status of rats with polycystic ovarian syndrome	Alwan and Al-saeed,, (2021)
19	*Salvia officinalis* leaf	Ag@NP	Anti-human ovarian cancer activity	Luo et al. (2022)
20	*Tilia cordata* flowers	Ag@NP	Antioxidant and anti-tumor activities	Saygi and Cacan, (2021)
21	*Aegle marmelos*	Chitosan entrapped Ag@NP	Human cervical cancer cells-HeLa	Sukumar et al. (2022)
22	*Curcumae kwangsiensis* leaf	Au@NP	Anti-human ovarian cancer	Chen et al. (2021)
23	*Spondias dulcis (*Anacardiaceae*)* peel	Au@NP	Breast cancer	Pechyen et al. (2022)
24	*Vigna radiata*	Au@NP	Inhibits colony formation in breast cancer	Singh et al. (2021)

### 3.7 Top groups working on nanoparticles for cancer therapy

**FIGURE 4:**
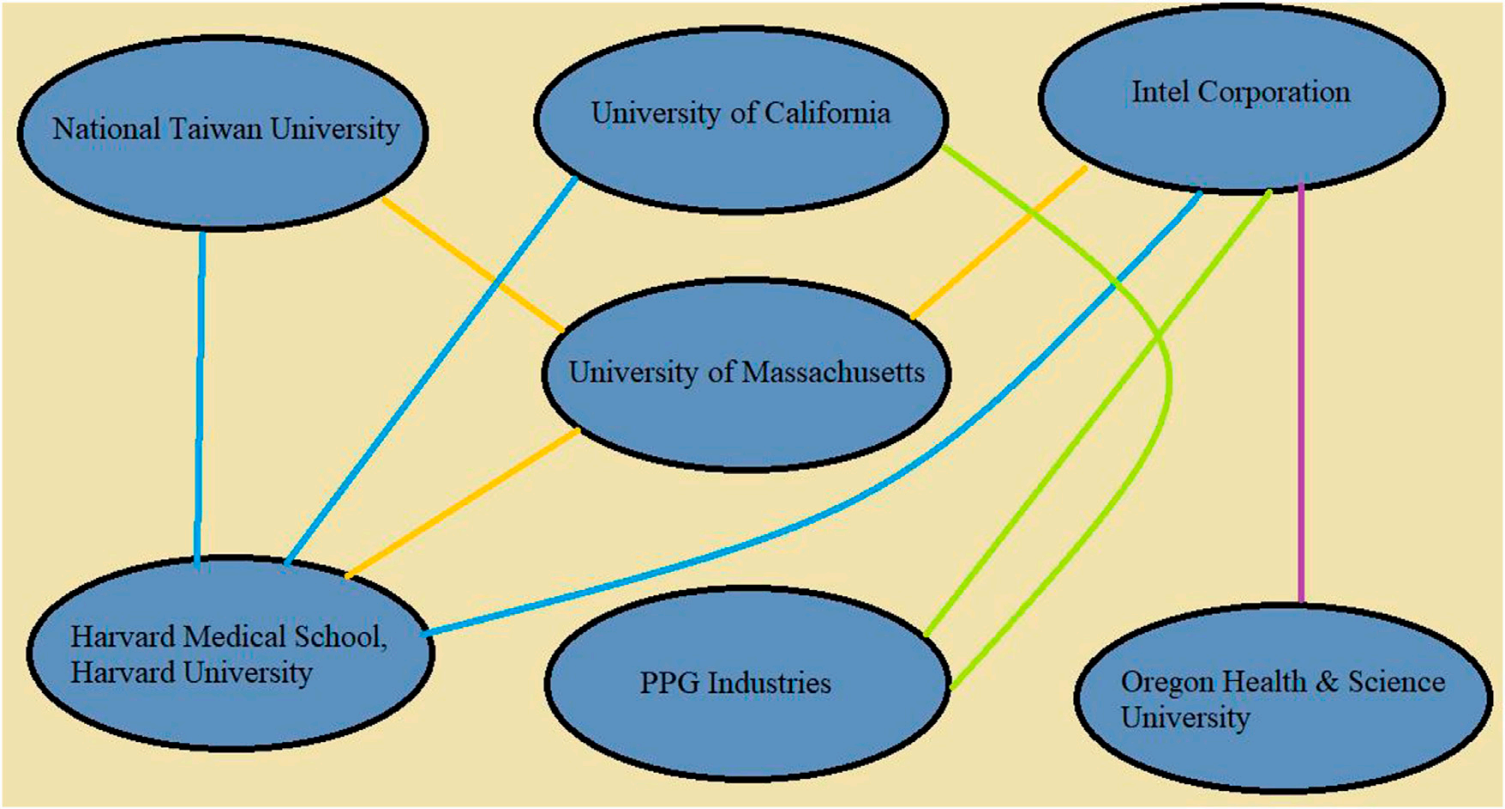
Interconnection between organizations for cancer therapy through use of nanoparticles.

Globally there are many potential research groups are working on nanoparticle cancer therapy. Table 4 shows the collaboration among top groups/institutes/research labs working on gold nanoparticles/silver nanoparticles/nanoparticles for treatment of cancer. Figure 4 represents the interconnection or collaborations between these groups. Such data enhanced the scope of bibliographic analysis. These findings reflect that researchers are working on nanoparticles for cancer therapy since long, but to overcome their toxic effect, now researchers are moving ahead towards use of biogenic nanoparticles.

**TABLE 4:** Details of groups working on cancer therapy using nanoparticles.

S.No.	Author	Affiliation	Collaborated with	Affiliation
1	Baoshan Xing	University of Massachusetts	Jian Zhao	Ocean University of China
2	Vincent Rotello	University of Massachusetts	Yi-Cheun Yeh	National Taiwan University
3	Yi-Cheun Yeh	National Taiwan University	Rubul Mout	Harvard Medical School, Harvard University
4	Yi-Cheun Yeh	National Taiwan University	Bradley Duncan	MIT Lincoln Laboratory, Harvard University
5	Yi-Cheun Yeh	National Taiwan University	Gulen Yesilbag Tonga	Harvard Medical School, Harvard University
6	Gulen Yesilbag Tonga	Harvard Medical School, Harvard University	Krishnendu Saha	Intel Corporation
7	Krishnendu Saha	Intel Corporation	Xiaoning Li	Oregon Health & Science University
8	Krishnendu Saha	Intel Corporation	Sung Tae Kim	Inje University
9	Krishnendu Saha	Intel Corporation	Daniel F. Moyano	PPG Industries
10	Krishnendu Saha	Intel Corporation	Sukru Gokhan Elci	University of Massachusetts
11	Ziwen Jiang	University of California	Daniel F. Moyano	PPG Industries
12	Ziwen Jiang	University of California	Gulen Yesilbag Tonga	Harvard Medical School, Harvard University

## 4 Obstacles in the journey of nanoparticles from clinic to market

Though first nano chemo drug Doxil entered the market in 1995, even after 27 years, number of nanomedicines approved by FDA are still inconsequential. 16 nano-based cancer drugs have been recommended by FDA whereas nearly 75 nano formulations are in clinical trials now (He et al., 2019). Still the gap between research and actual marketability of the drug molecule is huge. Some of the drugs even after approval are withdrawn from the market due to various reasons.

Major challenges associated with the use of NPs as drug for cancer are as follows:

### 4.1 Biological challenges

Nanoparticles are injected intravenously in to blood and they do not get enough time to interact with target site or we can say they lack time due to route followed for administration of drug (Lv et al., 2012). As a result, they are required in higher concentration to show the desirable impact. As people are using biologically safe NP’s but still in some cases it leads to organ failure such as lungs, liver etc., due to difference in solubility, particle size, surface area and other associated factors (Jia et al., 2018). According to some findings the utilization of controlled Ag@NPs prepared from extract of *Gongronema Latifoliumis* are beneficial as compared to conventional drugs, AgNPs have a larger inhibitory zone against bacterial cells (Aisida et al.,2019).

### 4.2 Technological challenges

Technological issues associated with nanoparticles are optimization and scale up of synthesis, as most of the synthesis are done at smaller scale and testing is mostly done *in vitro* level and sometimes *in vivo*. Clinical translation of NPs as drug for cancer therapy will be more feasible if *in vitro* and *in vivo* studies are also combined with computational and modelling studies based on understanding of mechanism of metastasis in cancer. (Gavas et al., 2021).

Cellular internalization of nano chemotherapeutic drug poses problem for its clinical development. Drug comes in to market once complete cellular internalization is defined. Also, response of each patient to the same drug is different due to change of genetic history and environmental factors. Furthermore, it takes very long span of time from drug development to pre-clinical and clinical trials on higher animals and humans (Mundekkad and Cho, 2022). Clustered Regularly Interspaced Short Palindromic Repeats (CRISPR) may offer an alternative for cancer cure and many nanoparticles have been used to produce targeted CRISPR/Cas9 *to stop the growth of tumor cells* (Shi et al., 2020; Duan et al., 2021).

## 5 Conclusion and future scope

Bibliographic analysis provides the record of publications with respect to input of keywords. This bibliometric analysis was done on biogenic silver and gold nanoparticles utilized in breast, ovarian and cervical cancer treatment from Clarivate Analytics Web of Science (WoS) and Scopus for the duration 2010 to 2022. The findings of bibliographic analysis are providing the information regarding the top publications, top journals in which maximum publications of this field are available and articles details which are having experiments on cell line. This review also provides the details of variety of plant sources which are utilized for the preparation of biogenic Au@NPs and Ag@NPs in different applications including cancer therapy, which is an additional support for bibliographic analysis. All such studies revealed the benefits of biogenic nanoparticles in various applications. The discussion on applications of NPs on cell lines in cancer therapy was also elaborate the efficacy of biogenic NPs against cancer. These reports reveal that though nanoparticles have been studied *in vitro* for anti-proliferative activity against cell line still *in vivo* research applications are very less and clinical translation of nanoparticles as medicines for cancer treatment is almost negligible. To design NPs which possess good solubility, specificity, non-toxicity, flexibility, binding capacity and can be utilized for target drug delivery systems, it is important to understand mechanism of formation of Protein Crown, enhanced permeability and retention effect, cellular and physiological factors that control effectiveness of NPs based drug delivery systems. After understanding biological and technical obstacles in the journey of Nanoparticles from clinic to market, it is a high time to intensify and further explore experimental studies on green nanoparticles to understand their mechanism of action at molecular stage. This article may help many researchers understand the flow of experiments on utilization of biogenic Au@NPs and Ag@NPs in cancer therapy, the gaps and the publication trends, to start off further experimentation that might lead to the drugs for the cancer treatment and also overcome the obstacles in this field.
